# Transferability of Military-Specific Cognitive Research to Military Training and Operations

**DOI:** 10.3389/fpsyg.2021.604803

**Published:** 2021-02-18

**Authors:** Christopher A. J. Vine, Stephen D. Myers, Sarah L. Coakley, Sam D. Blacker, Oliver R. Runswick

**Affiliations:** ^1^Occupational Performance Research Group, Institute of Sport, University of Chichester, Chichester, United Kingdom; ^2^Faculty of Sport, Allied Health and Performance Science, St Mary's University, Twickenham, London, United Kingdom; ^3^Department of Psychology, Institute of Psychiatry, Psychology & Neuroscience, Kings College London, London, United Kingdom

**Keywords:** occupation, environment, representative design, external validity, soldier, combat

## Introduction

**Figure 1:**
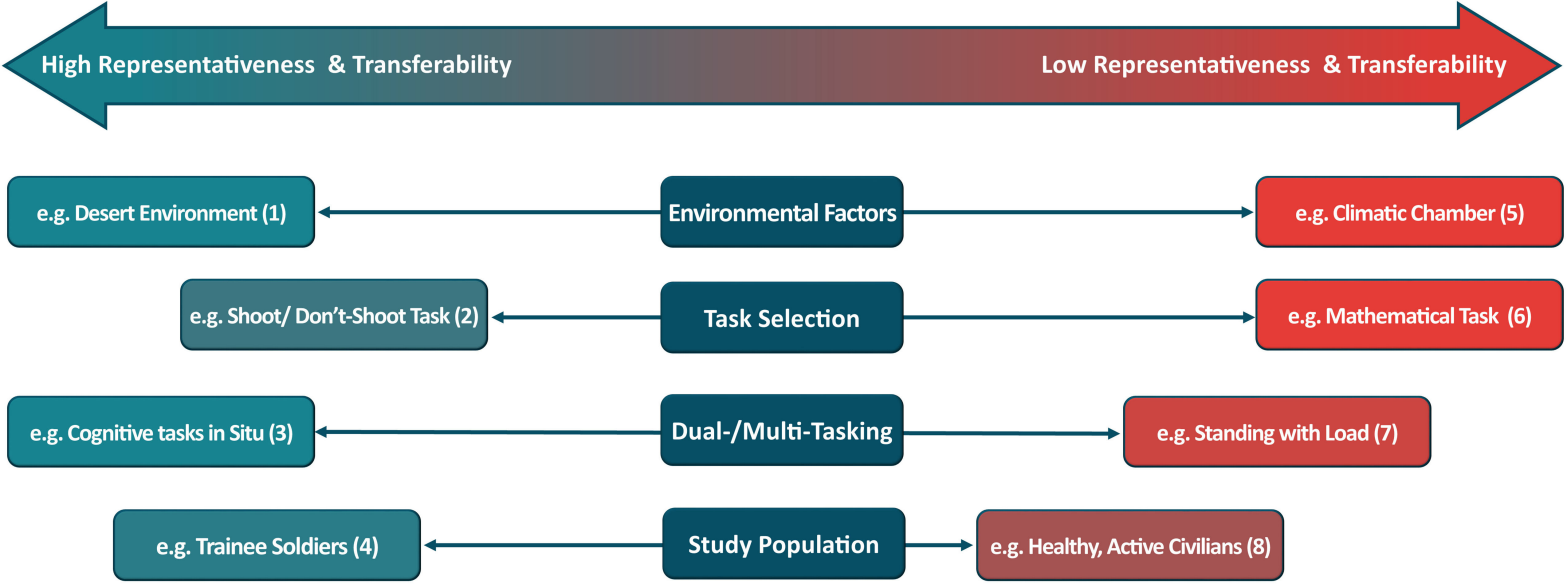
The continuum between high representativeness and high transferability to low representativeness and low transferability. Where numbers denote references for each example: (1) Bhattacharyya et al. (2017); (2) Kobus et al. (2010); (3) Giles et al. (2019); (4) May et al. (2009); (5) Caldwell et al. (2011); (6) Nibbeling et al. (2014); (7) Son et al. (2019); (8) Roberts and Cole (2013).

The influence of acute aerobic exercise on cognitive function is well documented (e.g., Lambourne and Tomporowski, 2010; Chang et al., 2012). However, the influence of military specific exercise on aspects of cognitive function relevant to military operations is less well understood. With the increasing physical and cognitive loads placed on military personnel (Mahoney et al., 2007), this interaction is fundamental to understanding operational performance (Russo et al., 2005). As such, ensuring the transferability of military-specific cognitive research to military training and operations, is of great importance, particularly for the development of both mitigation and enhancement strategies (see Brunyé et al., 2020). Despite this, studies have not always considered whether meaningful translations can be made. We suggest that researchers should endeavor to strike the balance between external validity and experimental control (Figure 1), and consider the concept of representative design (Pinder et al., 2011). External validity refers to the transferability of research findings from the research to the target population, whilst representative design refers to methodological approaches chosen to ensure that the experimental task constraints characterize those experienced during performance (i.e., the training or operational environment) (Pinder et al., 2011). Herein, we will focus on representative design during load carriage investigations, due to its mission criticality (Knapik and Reynolds, 2012), and it being the primary physical activity choice during military specific exercise-cognition research. Specifically, we discuss the inclusion of dual-/multi-tasking, implications of study population, cognitive task selection, and the data collection environment.

## Inclusion of Dual-/Multi-Tasking

The number of tasks presented, and when performance in these tasks is measured is crucial for representative design and external validity respectively. During operations, combatants are required to complete numerous physical and cognitive tasks concurrently; termed dual-/multi-tasking (Pellecchia, 2005). For example, during load carriage soldiers are required to simultaneously maintain situational awareness, whilst monitoring auditory and visual stimuli (Kobus et al., 2010). This additive effect increases cognitive demands; a result of task demands and the required coordination processes (Son et al., 2019). As such, the ability to manage the interference of, and switching between, conflicting tasks is of high importance during dual-/multi-task performance (Fallahtafti et al., 2020). Failure to do so can result in a performance decrement; termed the dual-task interference effect (Schmidt and Lee, 2013).

A number of load carriage focused studies, assessing cognitive function, have used a pre-/post-load carriage cognitive assessment methodology (Knapik et al., 1997; Bhattacharyya et al., 2017). Importantly, this pre-/post-load carriage methodology solely provides cognitive performance information at the instance of testing, and not during the load carriage tasks itself. This information during a load carriage task is of particular interest given that such tasks are often protracted in nature (e.g., 30 min to 18 h; Vine et al., 2017). The importance of within task assessment is evidenced by a number of studies. For example, Eddy et al. (2015), observed an increase in false alarms (auditory go/no-go task) in a loaded (40 kg) compared to an unloaded condition. However, across six time points, this only occurred in the third, fourth, and fifth. Similarly, Kobus et al. (2010) observed differences in percentage hit rate (detection and identification task) across all assessment time points in each of the three load conditions (0 vs. 45.5 vs. 61.2 kg). Whilst no pre-/post-load carriage comparisons were made in either study, Eddy et al. (2015) observed no difference between load conditions (0 vs. 40 kg) at either the first or last assessment point, suggesting differences could have been missed had a pre-/post-comparison been used. It has also been suggested that there is often sufficient recovery, post-physical task, for individuals to manage their cognitive resources, enabling the successful completion of the cognitive assessments (Mahoney et al., 2007). Finally, from a representative design perspective, military physical tasks are rarely discrete entities, and are undertaken with numerous interacting constraints and transitions between tasks. Therefore, within task measurements are of far more practical importance than those obtained once the task is complete. Consequently, where possible, it is key that studies undertake a dual-task approach, as they provide both more operationally relevant outcomes and provide greater granularity to the evidence base.

## Implications of Study Population

When considering the transfer of research findings to training and operations considerations should be given to study populations. Military personnel undergo extensive training and rehearsal to be able to execute their missions successfully (Nindl et al., 2013). Through these preparatory efforts, military specific exercise-cognition interaction effects are likely to be positively attenuated as a consequence of cognitive load reduction. Training will beneficially alter combatants' perceptions of factors including physical exertion, comfort, and task difficulty; in turn likely reducing cognitive load. For example, following heat adaptation, an individual's perception of physical exertion and thermal sensation, whilst exercising at high temperatures, are reduced (Tyler et al., 2016). Without this heat adaption, perceived exertion and thermal discomfort would increase, likely leading to irrelevant distractor processing, and a reduction in cognitive function (see Load Theory: Lavie et al., 2004; Lavie, 2010).

The interaction between cognitive assessment selection and study population is also likely to impact the subsequent outcomes, again by altering cognitive load. Specifically, whether the cognitive task completion requires either implicit or explicit processes is likely to impact the magnitude of performance change (Dietrich and Audiffren, 2011). Whilst, the distinction between these processes is greatly contested, and often more complex than assumed (De Houwer and Moors, 2007), broadly, the former relates to automated processing, whilst the latter refers to conscious processing. Therefore, with greater task familiarity, experienced personnel are likely to employ more automated processes compared with a novice, this is turn is likely to reduce the magnitude of possible performance attenuation (Martin et al., 2019).

Finally, a key critique of the exercise-cognition literature by McMorris (2016) relates to the inadequacies of reporting exercise intensities within studies. Previously, McMorris and Hale (2012), have suggested the use of low [< 40% maximal oxygen uptake (VO_2max_)], medium (≥40-<80% VO_2max_), and heavy (≥80% VO_2max_) domains for describing exercise intensities; which were adapted from Borer's (2003) categories. Importantly for exercise-cognition research, these boundaries were designed to coincide with key catecholamine and hypothalamic–pituitary–adrenal axis hormone thresholds. However, training status and testing modality are likely to influence the occurrence of these physiological thresholds relative to maximal capacities (e.g., VO_2max_ or maximum work rate) (Jamnick et al., 2020). Consequently, it appears that the use of physiological parameters, such as ventilatory and lactate thresholds are preferable compared with maximal capacities when describing exercise intensities (e.g., Podolin et al., 1991).

Collectively these factors highlight plausible differences between study populations. It is however important to note that access to military personnel can be difficult. In these cases, careful control of population characteristics (e.g., similar fitness levels) and ensuring thorough familiarization (both to the physical and cognitive tasks, along with clothing and protocols) is imperative for minimizing differences between novice and expert populations, and in turn ensuring the maximum transferability of findings. Moreover, whilst beyond the scope of this piece, it is important to also acknowledge that military performance is fundamentally a result of team performance (Shuffler et al., 2012; Billing et al., 2020), thus additional factors may impact performance outcomes beyond those investigated within individual based research (e.g., group cohesion).

## Cognitive Task Selection

When developing representative research paradigms, which aim to enhance transferability of findings, there is a need for clear consideration when selecting cognitive tasks. Within the military-specific exercise-cognition literature a variety of cognitive assessment approaches have been employed; from “basic” non-military specific-assessment (e.g., computer-based work tasks; Knapik et al., 1997; Bhattacharyya et al., 2017) to more externally valid military-specific assessments (e.g., military specific go-/no-go task; Eddy et al., 2015; Giles et al., 2019). With regards to “basic” non-military assessments, these typically isolate individual aspects of cognitive function, which differs from multicomponent requirements placed upon combatants during military operations (Vine et al., 2021). In addition, cognitive task selection is likely to have a direct impact on the magnitude and direction of a performance change. Therefore, it is crucial that the cognitive tasks selected match operational task demands. Moreover, whilst limitations to study size and task selection may exist, Vine et al. (2021) demonstrated poor to no correlation between “basic” and military-specific cognitive assessments. This suggests that either different cognitive processes are being assessed, or more likely, that the complexity of a military task requires numerous cognitive processes to be simultaneously executed. Further cementing the importance of opting for externally valid cognitive assessment methods.

When choosing a cognitive assessment, another factor to consider is the differing exercise-cognition responses for a given type of cognitive assessment. For example, in a meta-analysis by McMorris and Hale (2012), the authors highlighted differing effect sizes for exercise on speed and accuracy focused tasks. Critically, as both parameters are imperative for military operators, it is important to assess both during military-focused research. In addition to this, external validity can be enhanced by selecting cognitive tasks that would be concurrently completed during the physical task of choice. For example, the demands of a visual shoot/don't-shoot (Kobus et al., 2010; Armstrong et al., 2017) or audible go/no-go (Eddy et al., 2015; Armstrong et al., 2017; Giles et al., 2019) task reflect those that would be reasonable to expect during load carriage. Finally, due to the nature of military operations, physical taskings are rarely discrete in nature, but instead form a larger, more varied and often continuous work schedule. Due to repeatability being a limitation of representative design, quantifying the magnitude of both day-to-day and within-day variance, is a critical step in obtaining meaningful data in these scenarios. However, only a single study has reported the variance in performance of military-specific cognitive assessments (Vine et al., 2021). Collectively, these points demonstrate the importance of employing military-specific cognitive assessments in order to ensure the transferability of findings to military operations.

## Data Collection Environment

Combatants are required to operate effectively under a multitude of environmental constraints (e.g., mountainous, urban) with many of these providing additional challenges for military researchers. However, these additional environment specific stressors, highlight the importance of representative design given the likely interaction between these constraints and cognitive performance. Whilst safety and ethical implications of a “fully” representative military data collection environment make this an impractical approach, more representative designs can still be achieved. At a very simplistic level, soldier's must scan the oncoming terrain for hazards and obstacles in order to identify safe foot locations (Mahoney et al., 2007). This additional competition for cognitive resources, is inherently included within field-based investigations (Crowell et al., 1999; Nibbeling et al., 2014; Giles et al., 2019), but not typically applied during laboratory investigations. This laboratory research omission is despite data demonstrating a reduction in vigilance task performance, and an increase in distance covered by individuals (despite being able to step over them), when walking and avoiding obstacles (Mahoney et al., 2007). Similar results have also been observed when using monocular see-through head-mounted displays; whereby a dramatic reduction in a visual monitoring task was observed during walking, but not standing conditions (Mustonen et al., 2013), along with increased response times and reduced accuracy (Sampson, 1993).

Another consideration is the impact of thermal environmental conditions on cognitive performance (see review by Martin et al., 2019). Despite this comprehensive evidence, only two cognitively focused load carriage investigations have been conducted outside of normothermic conditions (Caldwell et al., 2011; Bhattacharyya et al., 2017). Importantly, many operational environments exist where a combination of environmental conditions may be apparent (e.g., altitude and cold). These conditions may have indirect effects, such as dehydration which has been shown to predict the decrement in central executive tasks and perceptions of mood state during exercise in the heat (McMorris et al., 2006). With both primary and secondary implications of environmental conditions, it emphasizes the importance of this factor within representative design.

Finally, during operations, combatants experience high levels of anxiety due to the constant threat of an enemy attack (Nibbeling et al., 2014). As with the other environmental considerations, the impact of anxiety is additive to the other cognitive challenges. Purportedly, anxiety will result in an attentional shift from task-relevant to task-irrelevant information; likely causing combatants to miss critical information (Nibbeling et al., 2014). Whilst a number of publications have detailed the relationship between anxiety and cognitive performance in police scenarios (e.g., Oudejans, 2008; Nieuwenhuys and Oudejans, 2010, 2011; Nieuwenhuys et al., 2012), considerably less attention has been given within the military sphere (Nibbeling et al., 2014). Again, highlighting the diversity and prevalence of interacting factors within the battlefield environment that may dramatically influence cognitive performance and further cementing the requirement for representative study designs. Moreover, we suggest, given the similarities between military, non-military uniformed services (e.g., emergency services), and other physically demanding occupations (e.g., mining and energy sectors) this approach should also be utilized with these populations.

## Conclusion

With a growing interest in the military-specific exercise-cognition relationship, it is key that observations can be translated from a research setting to military training and operations. Whilst some caveats pertaining to representative design exist, we encourage its further use within military research. In particular, we have shown that this can be achieved through an optimized balance between experimental control and external validity for the key parameters of dual-/multi-tasking, study population, cognitive task selection, and data collection environment.

## Author Contributions

CV wrote the initial manuscript draft. CV, SC, SM, SB, and OR then revised the manuscript collaboratively. All authors gave final approval for publication.

## Conflict of Interest

The authors declare that the research was conducted in the absence of any commercial or financial relationships that could be construed as a potential conflict of interest.
